# Impacts of early clinical exposure on undergraduate student professionalism—a qualitative study

**DOI:** 10.1186/s12909-022-03505-5

**Published:** 2022-06-06

**Authors:** Chun-i Liu, Kung-pei Tang, Yun-chu Wang, Chiung-hsuan Chiu

**Affiliations:** 1 Wan Fang Hospital, Taipei Medical University, Taipei, Taiwan; 2 Department of Early Childhood and Family Education, College of Education, National Taipei University of Education, Taipei, Taiwan; 3 Shuang Ho Hospital, Taipei Medical University, New Taipei City, Taiwan; 4 School of Health Care Administration, Taipei Medical University, Taipei, Taiwan

**Keywords:** Medical professionalism, Medical humanities coursework, Early clinical exposure, Undergraduate medical education

## Abstract

**Introduction:**

Early clinical exposure (ECE), or authentic human contact in a social or clinical context during preclinical training, has been adopted by many medical schools. This study aims to investigate how medical students’ sense of professionalism changed after ECE intervention, with the aim of informing curriculum design to enhance student awareness of the importance of medical professionalism.

**Method:**

Focus groups of ECE students were held to collect data for the study. All participants read interview guidelines before starting. During the focus groups, the students discussed their medical obligations as perceived throughout the course, which offered a choice between four different ECE tracks. They were then asked to report their understanding of the situations they encountered during the course and reflect on their implications.

**Results:**

Six focus groups of 22 students in total from a medical school in northern Taiwan were held shortly after the students completed an ECE course in September 2019. From their responses, 10 categories relating to medical professionalism were deduced categorized under 5 major dimensions. An additional 8 sub-dimensions on attitudes and 2 sub-dimensions on personal well-being were also identified as new categories separate from but related to medical professionalism. After the ECE intervention, about 59% of participants redefined their understanding of medical professionalism.

**Conclusion:**

ECE and intensive interaction with key stakeholders, including patients and their families, help students in the early stages of medical education form and cultivate a sense of medical professionalism. However, the relationship between participants’ personalities, motivations, and clinical activities requires further investigation.

## Introduction

It is difficult for medical educators to equip students with all the tools they will need in their future careers. Teachers have proposed a variety of models to teach the many competencies required of a doctor, such as the scientific approach or the biopsychosocial model [1]. However, it was not until recently that a variety of curricula have been developed to cultivate medical professionalism among medical students [2–6].

Medical professionalism plays an important role in guiding physicians as they make daily clinical decisions, practice ethics, and respond to societal expectations [7], yet its definition is difficult to pin down. Most definitions cover a range of perspectives, including values, attitudes, and behaviors [8, 9]. Some studies define medical professionalism as a virtue or moral [10–12], while others expand its scope beyond ethics [8, 12, 13]. Referencing these various frameworks, Chiu formulated 5 theoretical dimensions for medical professionalism, each further divided into 3–4 theoretical categories [8]. This framework was developed based on medical culture in Taiwan for use in investigating medical professionalism among plastic surgeons [9] and medical students [8, 14]. By including previous definitions [15], this conceptualization provides a holistic interpretation of medical professionalism.

Another challenge lies in transferring an understanding of medical professionalism from faculty to students. Some widely discussed approaches involve the reflective method [15], socialization, role models, service learning, or natural proposition [13], although one other has been gaining ground in recent years. Derived from the Flexner Report [1, 16–18], early clinical exposure (ECE), or authentic human contact in a social or clinical context during preclinical medical training, has been adopted by medical schools worldwide to close the gap between basic and clinical sciences. Since medical knowledge and professionalism are too intertwined to be learned in isolation [17], recent studies have shown that certain clinical practices have already been integrated into the early stages of medical education throughout the world [1, 16, 19–29]. While the term used to narrowly refer to formal clinical experiences early in medical school, ECE is based on a broad definition that includes community contact [30, 31] and early student-patient contact [32–38], as well as early clinical exposure [19, 20, 23, 29]. Reviews of ECE curricula have found that early exposures could consist of supervised clinical placements and sometimes direct exposure to patients, their families, and the community [31, 39]. ECE could therefore refer to a variety of clinical activities before official clerkships and internships.

Previous studies have documented the benefits of ECE [22, 40–43]. When incorporated into the medical sciences curriculum, ECE improved students’ academic performance [19, 21], while many studies have shown its benefit in helping students translate medical knowledge into clinical practice [1, 19, 39, 41, 44]. Studies have also proven the efficiency of ECE in building a sense of professionalism [13, 35, 39, 45]. As the beginning of professional socialization, ECE is usually paired with mentorship or role-model pedagogy [7, 46–49]. However, few studies investigate the effect of ECE on students’ own perception of medical professionalism.

Recent studies have identified factors that contribute to a successful ECE experience, suggesting that the structure of the ECE learning environment cannot be ignored [20, 33]. Nonetheless, little has been done to explore how different activities may influence students [26].

This study therefore aims to clarify: (1) How students perceive of their own professionalism before and after ECE intervention; and (2) What curricula best enhance student awareness of medical professionalism.

## Methods

### Curriculum design

This study focuses on a mandatory course entitled ‘Medicine & Society: Theory & Practice’ offered at a medical school in northern Taiwan. Designed based on ECE concepts, students can choose to take 1 of 4 modules (A, B, C, and D). Module A addresses situational learning in a clinical setting by arranging for students to observe and interview residents at a nursing station. Module B focuses on service learning in the community, namely constructing a dementia-friendly environment by interviewing healthcare providers and community members. Module C is held in an intensive care unit (ICU), and mainly deals with ethical issues regarding ventilator usage on terminally ill patients by interviewing caregivers and patients’ families. Module D emphasizes health promotion in local communities. Direct clinical exposure totals 4–6 h in each module, bookended by preparation beforehand and discussion with instructors afterward. After exposure, students are required to write a self-reflection their experience. About 160 first-year and second-year medical students enroll in the course each year.

### Study design

To understand how effective the ECE course is at fostering students’ sense of medical professionalism, this study held focus groups for students to discuss their experiences. Focus groups were conducted after students completed the course in September 2019. Before the focus groups began, all participants were expected to read the interview guidelines and reflect on their observations.

The interviewer was trained before conducting the focus groups to ensure the consistency and quality of data collected. Before obtaining informed consent from participants, the interviewer prior to the study explained its aims, participant confidentiality, and notified participants it would be audio recorded. Under the interview guidelines, all interviewees were first asked what they know about medical professionalism, then were invited to recall their experience of the course’s on-site component. They were then asked to describe at least one incident, conversation, or event that affected them the most while on site. The focus groups ended with the question: ‘To what extent did the clinical exposure improve your medical professionalism?’ Follow-up questions were asked to elicit more detailed descriptions of their experiences.

### Participants

Medical students who took the course during the spring of 2019 were eligible for inclusion. To cover all 4 tracks, 1–2 medical students from each track were invited to participate in each focus group. Participants were selected by convenience sampling and snowball sampling. Six focus groups were held with 3–5 students in each group, for a total of 22 participants.

### Data analysis

Audio files from each focus group were transcribed verbatim. Data were then analyzed using grounded theory, including inductive and deductive open coding [50]. The initial deductive analysis was coded independently by two researchers (CIL and YCW) based on the primary codes and aggregate dimensions developed by Chiu et al. [8]. All coders were required to prepare definitions and theories of medical professionalism before analyzing data. Coders initially formulated first-order codes that fit the original template, then proceed with inductive coding when new first-order codes emerged. New codes were reallocated through axial coding to new categories and dimensions. When there was a disagreement, a third investigator (CHC) was invited to discuss the codes and categories, and form a consensus to ensure that inter-rater reliability exceeded 0.8. Lastly, reciprocal checking was utilized to confirm that all first-order codes, categories, and dimensions were allocated correctly. The original template included 5 major dimensions and 14 categories. After coding, two more major dimensions on personal factors emerged in addition to the 5 major dimensions on medical professionalism, as well as 10 categories and 34 first-order codes.

### Ethical approval

This study was approved by the TMU-Joint Institutional Review (No. N201609020). The work was carried out in accordance with the Declaration of Helsinki. Students included in the study gave their oral and written informed consent. There was no potential harm to participants, and anonymity was maintained. All data and results are reported anonymously to ensure participant confidentiality.

## Results

### Descriptive statistics

A total of 22 students participated in the focus groups. Complete demographics and descriptive statistics are shown in Table 1. Of the participants, 41% (*n* = 9) were male and 86.4% (*n* = 19) were second-year medical students. Two participated in Module A (nursing station), 8 chose Module B (dementia ward), 4 were in Module C (ICU), and 8 participated in Module D (community). All of the participants were 19–22 years old.

**Table 1:** Participant demographics

**Demographics**	N (%)
Gender	
Male	9 (41%)
Female	13 (59%)
**Grade**	
First year	19 (86%)
Second year	3 (14%)
**Module**	
A: Nursing station	2 (9%)
B: Dementia ward	8 (36%)
C: ICU	4 (19%)
D: Community	8 (36%)

### Theoretical dimensions of medical professionalism

**Fig. 1:**
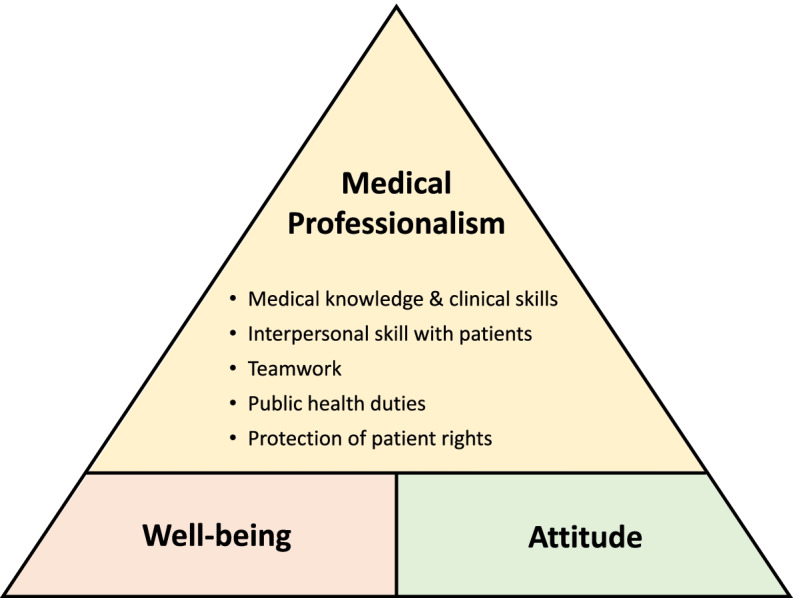
Dimensions of medical professionalism, predicated on student well-being and attitudes toward early clinical exposure

This study deduced 10 categories of responses based on the 5 major dimensions of medical professionalism as defined by Chiu et al. [8], in addition to 8 sub-dimensions on participant attitudes and 2 sub-dimensions on personal well-being. Associations between the three major dimensions are shown in Fig. 1.

The 5 dimensions of medical professionalism include medical knowledge and clinical skills (including continuous learning, professional identity, and professional reputation), interpersonal skill with patients (including compassion and communication), teamwork (including coordination and cooperation), public health duties (including health advocacy), and protection of patient rights (including information-sharing and self-determination, freedom to choose, and considering patients’ best interests). As all categories were deduced from the focus group transcripts and therefore differ slightly from existing definitions of medical professionalism [8], the categories discussed in the focus groups are detailed in Table 2.

**Table 2:** Self-reported levels of medical professionalism before/after ECE by theoretical category

Aggregate theoretical dimension	Theoretical category	Module A	Module B	Module C	Module D
**Medical knowledge & clinical skills**	Continuous learning		2/2		2/2
	Professional identity		1/1	1/1	1/1
	Professional reputation		2/2	1/1	
**Interpersonal skill with patients**	Compassion	1/1		2/3	1/1
	Equity				
	Integrity				
	Communication skills		3/4	2/4	4/4
**Teamwork**	Coordination & cooperation	0/1	0/1		1/2
	Knowledge-sharing				
**Public health duties**	Health advocacy				0/2
	Infection control				
**Protection of patient rights**	Information-sharing & self-determination				1/1
	Confidentiality & dignity				
	Freedom to choose				1/1
	Best interest of patients		0/2		0/1
**Attitudes**	Patience		0/2		2/2
	Congeniality		0/1		
	Scrutiny		1/2	1/1	
	Ingenuity		0/1		
	Emotional stability			1/2	
**Well-being**	Health/mental health	1/2	1/1	2/2	

Two new categories — attitude and well-being — emerged as items distinct from yet convergent with medical professionalism. ‘Attitude’ covers student patience and congeniality, scrutiny, ingenuity, and emotional stability. ‘Well-being’ covers the mental health and overall health/well-being of students. These two dimensions serve as the foundation on top of which medical professionalism may be built, and comprise the lessons participants learned from their clinical exposure. The new categories are described below in detail, including focus group quotations.

#### Compassion

Participants remarked on the importance of feeling another’s sorrow and standing in patients’ shoes as a basic quality necessary for interacting with patients and their families.

*‘Medical care personnel must have the ability to be compassionate to others' grief. If you don’t know how difficult they have it, you can’t face them with the appropriate attitude when they are emotional. If you just look at their anger and don't understand their grief, you can’t sympathize with them.’* (Case #E-3).

#### Communication skills

Participants reported learning to enhance their communication skills by fully comprehending a patient’s condition before approaching them and their family. The ability to comprehend and interpret lab results and recovery progress are critical to mutual understanding between physicians and patients.

*‘They [clinicians] mentioned that in doctor-patient communication, clinicians should summarize information rather than giving disorganized medical information. I feel that medical staff should communicate from the perspective of the family, rather than from the perspective of an expert.’* (Case #A-3).

#### Coordination and cooperation

Participants reported understanding the importance of teamwork after interviewing nurses. They also realized the mutual respect that nurses expect to have with physicians.

*‘I’ll never forget the nurse talking about cooperation with doctors, and how families deal with nurses. I think that nurses play an important role in medical work. They execute key aspects of patient care.’* (Case #A-1).

*‘They [nurses] have to put up with families and patients, some of whom respect them, but some don’t. I found that clinicians should not order the nurses around.’* (Case #A-1).

#### Health advocacy

Participants in Module B (dementia ward) reflected on their responsibility to promote public health through discussion of how the neighborhood near the hospital has managed to reduce traffic accidents. Some of them even mentioned their support for promoting disease prevention.

*‘If we could reduce traffic accidents, in a way, we are saving people. I thought that saving people was the same as saving their lives, but if we can prevent them from getting hurt, it also saves them.’* (Case #B-1).

#### Patients’ best interests

From visiting a dementia ward and the surrounding community, Module B participants reported learning holistic care that not only offers medical comfort, but also specialized facilities, and economic and social support.

*‘In addition to solving clinical problems, clinicians should provide comfort to patients. That means not only dealing with pain and disease, but also helping improve their environment and access to economic support.’* (Case #A-3).

#### Patience and congeniality

Participants in Module B said that patience was essential to being a good physician after visiting a dementia ward and observing how a neurologist interviewed his patients.

*‘The clinician remained calm and patient when the patient repeated a persecutory delusion that her caregiver was going to poison her.’* (Case #E-2).

Others cited congeniality as an important quality after shadowing attending physicians doing rounds.

*‘The clinicians knew details about their patients. They could point out what their patients remember or forget. It made me realize that clinicians are very responsible. They really care for every patient.’* (Case #A-2).

#### Scrutiny

One participant in Module B noted the design of the dementia ward, remarking that the touches of nostalgia could benefit patients with anterograde amnesia. He also highlighted the importance of empathic thinking.

*‘The nostalgic design of the dementia ward was cute, like the red brick wall. After the course, I learned that design can make patients feel more comfortable.’* (Case #A-3).

*‘Overall, I believe that clinicians should always think about reasons and details. In the medical field, there are reasons behind certain designs.’* (Case #A-3).

#### Ingenuity

A clinician’s encouragement to think outside the box and learn to stand in their patients’ shoes led one participant to remark on how innovative thinking that centers the patient is essential for physicians.

*‘Teachers encouraged us to think about what we can do for our patients. Before the *exposure*, we only learned in the classroom, but the teacher encouraged us to innovate and think outside the box.’* (Case #A-2).

#### Emotional stability

After interviewing patients’ families, participants reported the importance of managing their emotions and remaining neutral, regardless of a patient’s condition.

‘Clinicians should practice to make sure their clinical work isn’t affected by emotion. That means you can’t always be sad in your everyday work.’ (Case #E-3).

‘Resilience. A doctor should be resilient.’ (Case #D-1).

#### Well-being

Through interviews with clinicians, participants noticed the importance of balancing one’s own health with patient care.

‘The clinician emphasized the importance of relaxation. Even when you are on duty, you should know how to relax. You can’t solve any problems when you’re exhausted.’ (Case #D-2).

‘Stress management. Well-being.’ (Case #E-3).

### ECE effects

**Fig. 2:**
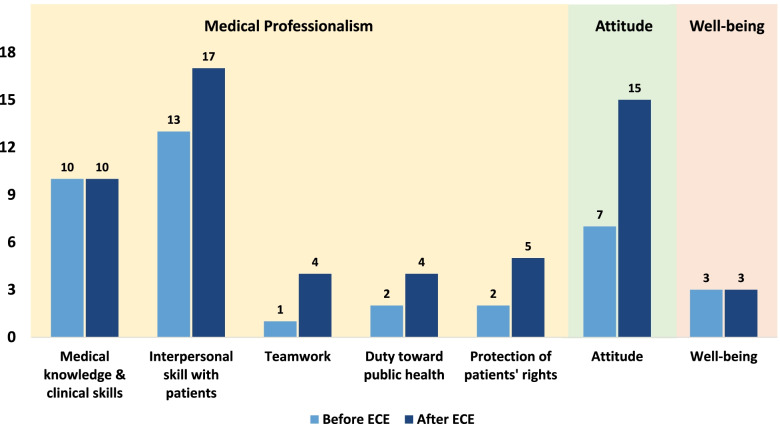
Mentions of physician qualities and expectations before and after intervention

Participant responses from before and after ECE were organized and tallied according to the 5 sub-dimensions of medical professionalism, as well as the additional dimensions of attitude and well-being identified after intervention (Table 2). Before ECE, ‘medical knowledge and clinical skills’ and ‘communication skills’ were the most frequently mentioned sub-dimensions, while ‘teamwork,’ ‘public health,’ and ‘protection of patients’ rights’ were rarely mentioned. After ECE, about 59% of participants said they redefined their understanding of medical professionalism. About 13.6% changed their attitude to embrace ‘communication skills,’ while another 13.6% added ‘protection of patients' rights’ to their definition. Moreover, other emergent dimensions, such as thoughtfulness, patience, and personal well-being, were deuced after ECE. Figure 2 shows changes in participants’ recognition of medical professionalism before and after the intervention.

Further investigation of differences among the four modules reveals that participants of Modules B, C, and D supplied more insights on medical professionalism than those who chose Module A. This might be related to the design of these modules, as they incorporated intensive interaction between participants, medical staff, and third parties such as patients, patients’ families, and community residents, as opposed to only interaction with medical staff. Learning from direct encounters with patients and their families inspired participants to enhance their medical professionalism more than those who only interacted with medical staff. Designers of ECE interventions should take this discrepancy into account.

## Discussion

Before ECE intervention, most of the participants assumed that medical professionalism was limited to medical knowledge and communication skills. After the course, most participants added to this definition responsibility to promote public health and protection of patient rights.

Unlike our previous survey [8], this study reflects Taiwanese medical students’ personal understanding of medical professionalism. Before ECE, participants in 3 of the 4 modules viewed medical knowledge and clinical skills as core components of medical professionalism. This finding could be attributed to the education system in Taiwan and extreme emphasis on performing well on knowledge tests in high school. Since Asian cultures generally emphasize academic competitiveness, humanities or communication skills are often ignored.

Secondly, personality traits should be considered while developing ECE activities. Participants reported different takeaways from the same courses, for instance, one student after visiting a dementia ward noted the importance of considering patients’ best interests, while another keyed in on patience as a quality central to being a good clinician. The reason why ECE could influence students in such different ways could be attributed to their individual personalities. The diversity of activities in each of the four modules may have also influenced the participants’ awareness of different facets of professionalism. ECE is therefore a remarkably efficient and personal way of cultivating professionalism, and should be considered by course designers.

The four separate modules also triggered certain ways of understanding medical professionalism. In Module A for example, participants noticed ‘teamwork’ and ‘well-being,’ but did not mention ‘communication skills’ and ‘protection of patient right.’ This might be explained by the limited observation time and range of interests represented through interviews with physicians. Gibson’s dimensional framework provides a closer look at how role modeling functioned in ECE activities [51]. Role modeling seemed to be a neutral way to shape medical professionalism, but among those in Module B, there was no mention of ‘duty toward public health,’ although they did mention ‘medical knowledge and clinical skills’ and ‘well-being.’ In Module C, participants covered most dimensions, but not ‘teamwork,’ ‘duty toward public health,’ and ‘protection of patient rights,’ while in Module D, ‘well-being’ was not covered. Based on these results, service learning in a dementia ward (Module B), discussions of ethics in the ICU (Module C), and participating in a community health program (Module D) were most effective at shaping a comprehensive understanding of medical professionalism.

As in previous studies [16, 27, 28, 39], this study proved that ECE helps medical students cultivate their skills and professionalism. However, not all of the participants believed that the intervention helped further their skill as medical professionals. Half of the participants reported having an unsatisfactory experience, while none reported that ECE improved their motivation to perform well academically. This result is inconsistent with previous studies [19, 20, 23, 27, 29]. Since there are few studies exploring the impact of ECE on professionalism, it is hard to compare this finding.

As each module included many different activities, different elements may have affected participants in different ways. For example, visitors to the dementia ward observed interviews between neurologists and patients, a brief introduction to a clinician, and the design of the ward, and were invited to ask questions of clinicians. All of these elements may have separate effects on students’ awareness of professionalism, requiring further research to clarify their actual effects. Motivation and personalities of the participants also appeared to have significant bearing on learning outcomes, requiring more qualitative research to identify interactions between these factors.

As opposed to the definitions of medical professionalism proposed by Cruss [7], Castellani and Hafferty [52], and the Accreditation Council for Graduate Medical Education (ACGME) [53], which focus on Western culture contexts, the framework in this study was chosen because it reflects Taiwanese cultural values toward medicine [8]. This study therefore excludes certain categories from the five dimensions and deduces two additional categories, attitudes and well-being, that support medical professionalism. Missing categories might imply that participants did not identify them when reflecting on their course. Moreover, the emergence of well-being as a foundational element of professionalism might be explained by a rising awareness of personal wellness among younger generations.

### Limitations

The study has three major limitations. Firstly, due to time limits, each module included only 4–6 h of clinical exposure and 8–10 h of in-depth discussion with instructors. Cultivating professionalism is not a one-time intervention, but a long-term process. We therefore look forward to conducting a follow-up study investigating longer-term changes among the participants. Secondly, we did not address instructor skill and its potential effect on outcomes. Thirdly, there were a few topics that emerged in the focus groups that did not fit into our original framework [8]. Lastly, this result may only be applied to observable attributes of medical professionalism.

## Conclusion

ECE helps to form and cultivate medical professionalism in the early stages of medical education, although the relationship between participants’ personalities, motivation, and clinical elements should be taken into account in further research.

## Data Availability

No data have been submitted to any open-access databases. All data supporting the study are presented in the manuscript or are available from corresponding author upon reasonable request.

## Abbreviations

ECE: Early clinical exposure
ICU: Intensive care unit

## References

[1] Basak O (2009). Early clinical exposure in medical curricula across Europe: an overview. Eur J Gen Pract.

[2] Wear D, Castellani B (2000). The development of professionalism: curriculum matters. Acad Med.

[3] Birden H (2013). Teaching professionalism in medical education: a Best Evidence Medical Education (BEME) systematic review. BEME Guide No. 25. Med Teach.

[4] Zare S, Yamani N, Changiz T (2019). How to develop an undergraduate medical professionalism curriculum: Experts' perception and suggestion. J Adv Med Educ Prof.

[5] Huang CD (2021). How does narrative medicine impact medical trainees' learning of professionalism? A qualitative study. BMC Med Educ.

[6] Bashir A, McTaggart IJ (2022). Importance of faculty role modelling for teaching professionalism to medical students: individual versus institutional responsibility. J Taibah Univ Med Sci.

[7] Cruess RL (2014). Reframing medical education to support professional identity formation. Acad Med.

[8] Chiu C-H (2010). A professionalism survey of medical students in Taiwan. J Exp Clin Med.

[9] Chiu C, Pan S, Lin Y (2019). How plastic surgeons value professionalism: using q methodology to explore the prioritization of professionalism. Aesthetic Surg J.

[10] Brody H, Doukas D (2014). Professionalism: a framework to guide medical education. Med Educ.

[11] Branch WT (2000). Supporting the moral development of medical students. J Gen Intern Med.

[12] Irby DM, Hamstra SJ (2016). Parting the clouds: three professionalism frameworks in medical education. Acad Med.

[13] Tsai M (2019). Early exposure to global health raises self-awareness of medical novices on professionalism. J Med Educ.

[14] Chiu C, Wu J, Chen C (2019). Why do young physicians make a 'detour' to aesthetic clinics? An exploration of professional identity among young physicians who changed career paths. J Med Educ.

[15] Bryan CS, Babelay AM (2009). Building character: a model for reflective practice. Acad Med.

[16] Verma M (2016). Early clinical exposure: New paradigm in Medical and Dental Education. Contemp Clin Dent.

[17] Dornan T (2005). Osler, Flexner, apprenticeship and 'the new medical education'. J R Soc Med.

[18] Souza R, Sansevero A (2015). Introducing early clinical experience in the curriculum.

[19] Sathishkumar S (2007). Attitude of medical students towards early clinical exposure in learning endocrine physiology. BMC Med Educ.

[20] Tang KP (2019). Correlation between early clinical exposure environment, attitudes toward basic medicine, and medical students' basic science learning performance. BMC Med Educ.

[21] Kar M (2017). Early clinical exposure as a learning tool to teach neuroanatomy for first year MBBS students. Int J Appl Basic Med Res.

[22] Eika B (2001). Early clinical exposure–an instant success. The new medical curriculum at the University of Aarhus. Ugeskrift Laeger.

[23] Gupta K, Gill G, Mahajan R (2020). Introduction and Implementation of early clinical exposure in undergraduate medical training to enhance learning. Int J Appl Basic Med Res.

[24] Khabaz Mafinejad M (2016). Medical students' attitudes towards early clinical exposure in Iran. Int J Med Educ.

[25] Hopayian K, Howe A, Dagley V (2007). A survey of UK medical schools' arrangements for early patient contact. Med Teach.

[26] Abramovitch H (2002). A tale of two exposures: a comparison of two approaches to early clinical exposure. Educ Health (Abingdon).

[27] Dornan T, Bundy C (2004). What can experience add to early medical education? Consensus survey. BMJ.

[28] Lie D (2006). What do medical students learn from early clinical experiences (ECE)?. Med Teach.

[29] Rani MA, Sharma KS, Koirala S (2002). What do students say about the early clinical exposure at B.P. Koirala Institute of Health Sciences, Nepal?. Med Teach.

[30] Wilkinson T, Gower S, Sainsbury R (2002). The earlier, the better: The effect of early community contact on the attitudes of medical students to older people. Med Educ.

[31] Hannay D, Mitchell C, Chung MC (2003). The development and evaluation of a community attachment scheme for first-year medical students. Med Teach.

[32] Diemers AD (2007). Students' perceptions of early patient encounters in a PBL curriculum: a first evaluation of the Maastricht experience. Med Teach.

[33] Ottenheijm RP (2008). Early student-patient contacts in general practice: an approach based on educational principles. Med Teach.

[34] Miettola J, Mantyselka P, Vaskilampi T (2005). Doctor-patient interaction in Finnish primary health care as perceived by first year medical students. BMC Med Educ.

[35] Howe A (2007). Patient contact in the first year of basic medical training–feasible, educational, acceptable?. Med Teach.

[36] Vieira JE, do PatrocínioTenórioNunes M, de Arruda Martins M (2003). Directing student response to early patient contact by questionnaire. Med Educ.

[37] Haffling AC, Håkansson A, Hagander B (2001). Early patient contact in primary care: a new challenge. Med Educ.

[38] Forster DP (1992). The family study: a model for integrating the individual and community perspective in medical education. Med Educ.

[39] Dornan T (2006). How can experience in clinical and community settings contribute to early medical education? A BEME systematic review. Med Teach.

[40] Littlewood S (2005). Early practical experience and the social responsiveness of clinical education: systematic review. BMJ.

[41] McLean M (2004). Sometimes we do get it right! Early clinical contact is a rewarding experience. Educ Health (Abingdon).

[42] Shibli-Rahhal A (2019). A practical approach to integrating communication skills and early clinical experience into the preclinical medical school curriculum. Med Sci Educ.

[43] Chen H (2016). Students’ goal orientations, perceptions of early clinical experiences and learning outcomes. Med Educ.

[44] Duban S (1982). Teaching clinical skills to pre-clinical medical students: integration with basic science learning. Med Educ.

[45] Hellquist G (2005). Early professional contact supports professional development of medical students. EPC–a new course in medical education in Gothenburg. Lakartidningen.

[46] Mann MP (1994). A Light at the End of the Tunnel: The Impact of Early Clinical Experiences on Medical Students.

[47] Rooks L, Watson RT, Harris JO (2001). A primary care preceptorship for first-year medical students coordinated by an Area Health Education Center program: a six-year review. Acad Med.

[48] Woolliscroft JO, Schwenk TL (1989). Teaching and learning in the ambulatory setting. Acad Med.

[49] Ramachandran K (2015). Early clinical exposure through innovative interactive clinical anatomy lectures. Natl Med J India.

[50] Watling CJ, Lingard L (2012). Grounded theory in medical education research: AMEE Guide No 70. Medical Teach.

[51] Gibson DE (2004). Role models in career development: new directions for theory and research. J Vocat Behav.

[52] Castellani B, Hafferty FW (2006). The complexities of medical professionalism. Professionalism in medicine.

[53] ACGME (2022). The Accreditation Council for Graduate Medical Education.

